# Xanthogranulomatous endometritis: A case report and literature review

**DOI:** 10.1002/ccr3.4299

**Published:** 2021-06-24

**Authors:** Philippe Merviel, Pandora James, Marianne Carlier, Isabelle Thomas‐Kergastel, Marine Guilloique, Virginie Conan‐Charlet, Clara Bastard, Pascale Marcorelles, Yannick Jobic, Pierre‐François Dupré

**Affiliations:** ^1^ Gynecology and Obstetrics Department Brest University Hospital Brest France; ^2^ IFR 148 ScInBioS–EA 3878–GETBO Brest University Hospital Boulevard Tanguy Prigent Brest France; ^3^ Radiology Department Brest University Hospital Brest France; ^4^ Histopathology Department Brest University Hospital Brest France; ^5^ Cardiology Department Brest University Hospital Boulevard Tanguy Prigent Brest France; ^6^ INSERM UMR 1078 Medical University of Western Brittany Brest France

**Keywords:** endometritis, histopathology, magnetic resonance imaging, pyometra, xanthogranulomatous

## Abstract

Xanthogranulomatous endometritis is a rare benign pathology mimicking endometrial carcinoma.

## Graphical abstract

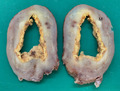

## 1 INTRODUCTION

Xanthogranulomatous endometritis is an unusual pathological entity mimicking endometrial carcinoma. We report a case of 87‐year‐old woman with an enlarged uterus without other clinical signs. After hysteroscopy and evacuation of 500 mL of lactescent fluid, the relapse of the collection led to a hysterectomy.

Xanthogranulomatous inflammation (XGI) is an exceptional condition implicating the presence of foamy histiocytes, and other inflammation cells affecting various organs, mainly the kidney and gallbladder.^1^ In the female genital tract, XGI has been observed in the form of endometritis and tubo‐ovarian abscess.^2^, ^3^ Xanthogranulomatous endometritis (XGE) is a rare benign endometrial pathology with few reported cases. Resemblance with endometrial malignancy is frequent making the diagnosis difficult, and therefore, attentive histopathological analysis is required.^4^, ^5^ We reported a case of a XGE and described the clinical signs, histopathologic characteristics, pathogenesis, treatments, and evolution.

## 2 CASE REPORT

**FIGURE 1:**
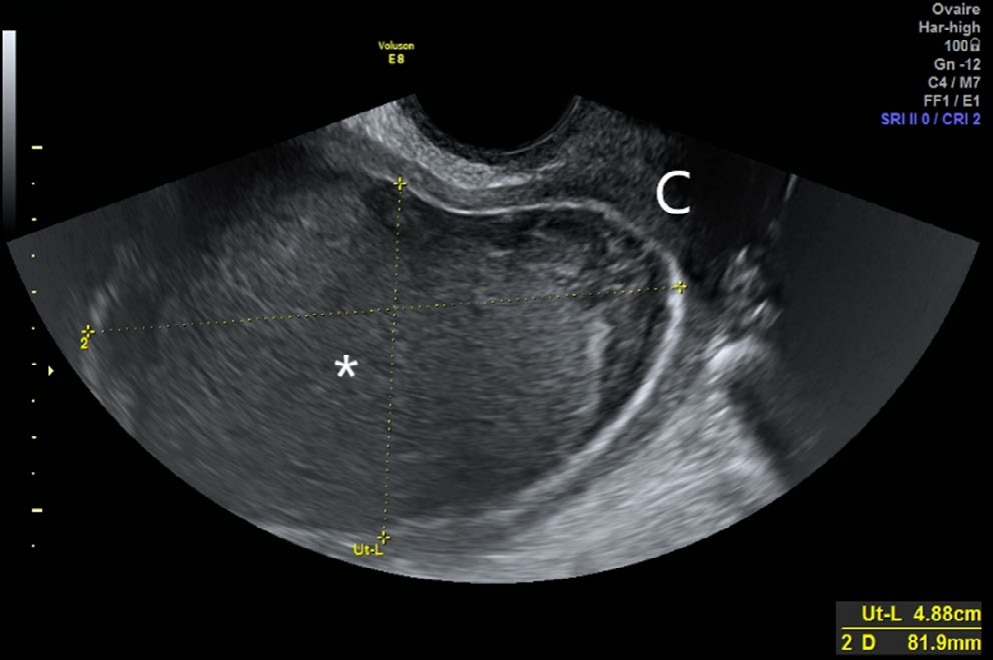
Pelvic ultrasonography sagittal view reveals an intrauterine cystic mass (81.9 mm length), suggesting hematometra or pyometra (*). C, uterine cervix

**FIGURE 2:**
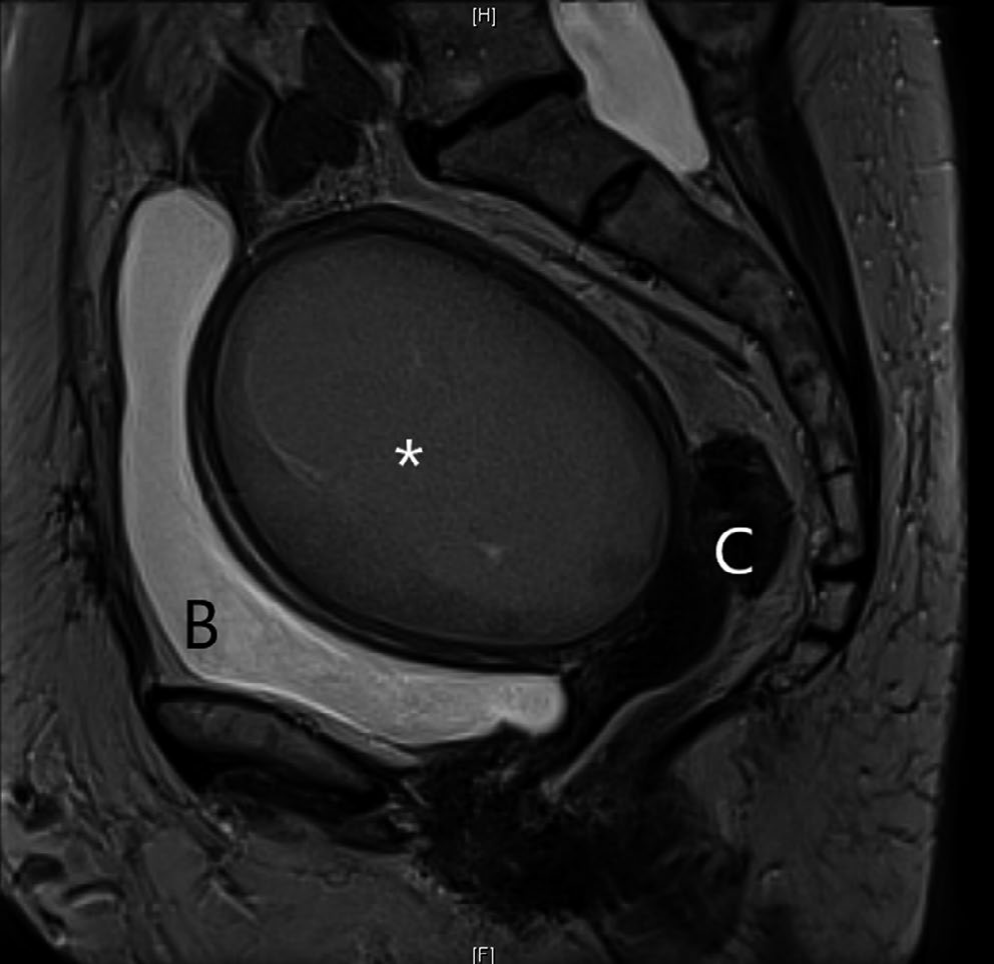
Magnetic resonance imaging of the pelvis (T2 sequence) revealed an enlarged uterus containing an intrauterine cystic mass (105 mm length), suggesting a pyometra (*). B, bladder; C, uterine cervix

A 87‐year‐old postmenopausal woman consulted as part of her annual gynecological follow‐up. Her medical‐surgical history includes bilateral breast in‐situ cancer treated by bilateral mastectomy, permanent atrial fibrillation, and a transient ischemic attack. She also carries an aortic bioprothesis. She had no history of endometriosis, pelvic inflammatory disease or use of intrauterine device. She denied any symptom of pelvic pain, fever, vaginal bleeding, or weight loss. Gynecological examination reveals an enlarged uterus for which a pelvic ultrasound is requested. The pelvic ultrasound (Figure 1) shows an intrauterine cystic mass of 82 mm length, with homogeneous internal echoes, suggesting hematometra/pyometra. Endometrial thickness was not increased and regular. A pelvic magnetic resonance imaging (MRI) (Figure 2) is performed and reveals a heterogeneous intrauterine collection measuring 105 mm of antero‐posterior axis suggesting pyometra. The myometrium is normal, and there is no enhanced tissue portion. No adnexal abnormality or digestive fistula was detected. The hematological (red and white blood cells), biochemical (hepatic and renal functions, C reactive protein), and neoplastic markers (CEA, CA 125, and CA19‐9) investigations were within the normal limits. Drainage of the fluid was attempted during speculum examination, but could not performed because of complete stenosis of the cervix. In view of cervical stenosis and pyometra in an elderly woman, a clinical possibility of endocervical or endometrial carcinoma could not be ruled out.

An operative hysteroscopy is performed under general anesthesia and after dilatation of the cervix visualizes an intrauterine lactescent liquid, which suggests pyometra. This liquid is odorless and could in fact correspond to noninfected blood in extreme degradation. The intervention enables the evacuation of 500 mL of liquid with a surgical aspiration device. Hysteroscopy shows no signs of malignancy, and the endometrium is not hypertrophic. An endometrial curettage is performed, and the histopathological analysis is normal. Bacteriological analysis of the fluid finds multisensitive Pseudomonas aeruginosa for which an antibiotic treatment (ceftazidime intravenous then ciprofloxacine per‐os) is administered. Biologically, there is no inflammatory syndrome and there is no argument for a bacterial infection.

**FIGURE 3:**
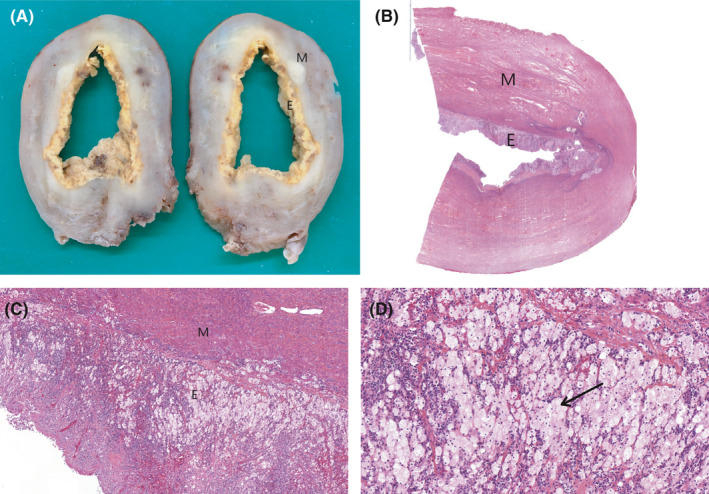
Histopathological analysis of the hysterectomy (E: endometrium; M: myometrium). A, Macroscopic view of the uterus with xanthogranulomatous endometritis. B, Microscopic photograph of the uterus in hematoxylin‐eosin‐saffron staining, at X1 magnification. C, Microscopic photograph of the endometrium and myometrium in hematoxylin‐eosin‐saffron staining, at X20 magnification. D, Microscopic photograph of the endometrium in hematoxylin‐eosin‐saffron staining, at ×40 magnification. Black arrow: foamy histiocytes

At the postoperative visit one month later, the pelvic ultrasound showed a reconstitution of the intrauterine fluid measuring 70 mm long. A total hysterectomy and bilateral salpingooophorectomy are performed. Standard histological technique was performed. The part was fixed in 4% buffered formalin (with a fixation time of 24 hours). The area of interest was included as a block. The latter underwent dehydration in successive alcohol baths of increasing concentration. The sample was then embedded in paraffin. Three μm sections with a microtome were made, then spread on a slide, and stained with hematoxylin‐eosine‐saffron. We observed an entirely destroyed endometrium, replaced by a polymorphic inflammatory infiltrate containing mainly plasma cells, as well as lymphocytes and some neutrophils. There are clusters of large cells with clear cytoplasm corresponding to foamy histiocytes. Some rare giant cells organized around cholesterol crystals are visualized (Figure 3). The histopathological analysis finally reveals an atrophic cervix and a dilated uterine cavity with necrotic endometrium consistent with intense lesions of xanthogranulomatous endometritis. Bilateral adnexa were atrophic with no evidence of inflammation, and peritoneal fluid was normal.

## 3 DISCUSSION

Xanthogranulomatous endometritis is a rare disease characterized by chronic inflammation of the endometrium. The first case of XGE was reported by Barua et al in 1978,^6^ and until this date <30 cases have been reported in the worldwide literature. The age of onset is from 59 to 88 years, with a mean of 72 y.o. Bleeding, excessive vaginal discharge, and cervical stenosis with or without pyometra are the most common symptoms.^7^

The pathogenesis of XGE is still incompletely understood. The cervical stenosis, as in our case, lead to the accumulation of endometrial secretions, infection, and tissue necrosis, and further release of cholesterol and other lipids which were phagocytozed by macrophages resulting in the formation of foam cells and XGE.^2^ In a number of women, diabetes mellitus has been found to be a possible risk factor for the development of xanthogranulomatous inflammation.^5^ Necrosis of tumor cells following irradiation in cervical or endometrial carcinoma can also induce chronic inflammation with pyometra.^8^ Histopathological examination of XGE is characterized by focal or total replacement of the endometrium by granulation tissue with presence of foamy histiocytes, variable numbers of multinucleated giant cells, chronic inflammatory cells, necrotic material, siderophages, calcium, and hemosiderin.^9^ The infiltration of the myometrium by foamy macrophages might be misdiagnosed as clear cell carcinoma or sarcoma, but the cytological characteristic should lead to the XGE diagnosis. Immunohistochemistry proves to be helpful in the diagnosis of XGE in difficult cases. The presence of CD68 positive foamy histiocytes and a chronic lymphocytic infiltrate positive for CD3 and CD20 is in favor of an inflammatory process over carcinoma. The absence of concentric calcific bodies (Michaelis‐Gutmann bodies) and negative α1 antitrypsin staining of foamy histiocytes is helpful to exclude a malakoplakia,^10^ the other differential diagnosis. Different bacteria, as Proteus vulgaris or Escherichia Coli, have been recognized as a contributing factor.^11^ In our case, a Pseudomonas aeruginosa was isolated in the intrauterine fluid. In some cases, severe atherosclerosis was observed in the uterine wall arteries, leading to ischemia which contributes to the development of XGE. Synonyms used for XGE include histiocytic endometritis and pseudoxanthomatous endometritis.^11^, ^12^

Malignancy forms are an important differential diagnosis of this lesion. XGE can be mistaken for malignancy on clinical and radiological appearance,^1^, ^13^ and cases of coexisting endometrial malignancy and XGE have been reported.^2^, ^8^ Russak et al ^2^ reported six cases of XGE associated with endometrial adenocarcinoma that had been irradiated prior to surgery. Contributory factors to the development of XGE have been reported such as necrosis, hemorrhage, obstruction, tumor bulk, or delay in treatment. Sampling of the entire endometrium appears to be the best option to eliminate that risk.^1^, ^4^

Recovery after antibiotic treatment or spontaneous resolution is the most frequent outcomes,^2^, ^14^, ^15^ but relapse is possible and radical surgery is then the appropriate treatment, as in this case. Lack of treatment seems to lead to a risk of systemic inflammation. One case of death directly related to XGE has been reported in a 68 years old woman with a history of abdominal pain. Death was caused by heart failure due to systemic inflammation, and pathological examination revealed XGE with transmural extension into the peritoneal cavity.^16^ In our case, despite the presence of Pseudomonas aeruginosa in 500 mL of fluid, peritoneal infection, or systemic infection did not occur.

## 4 CONCLUSIONS

We report a case of recurrent xanthogranulomatous endometritis with a major intrauterine retention of infected fluid. Since XGE may mimic cervical or endometrial carcinoma clinically and pathologically, knowledge of this unusual and rare inflammatory disease is important for gynecologists, radiologists, and pathologists.

## CONFLICT OF INTEREST

The authors report no conflicts of interest in relation to the present study.

## AUTHOR CONTRIBUTIONS

Philippe Merviel: substantially contributed to the conception, design of the work, the acquisition, analysis, and interpretation of clinical, ultrasound, and MRI data, and have drafted the work or substantively revised it. Pandora James: acquired the clinical data and translated the manuscript. Marianne Carlier: acquired the clinical data. Isabelle Thomas‐Kergastel: served as the head of the Women's Radiology department. Marine Guilloique: acquired the data on MRI. Virginie Conan‐Charlet: served as the Pathologist referent in this case. Clara Bastard: served as the Pathologist in this case. Pascale Marcorelles: served as the head of the Histopathology department. Yannick Jobic: acquired the clinical data. Pierre‐François Dupré: substantially contributed to the conception, the acquisition, analysis, and interpretation of clinical data.

## ETHICAL APPROVAL

The consent of the woman is available in the medical record and with the corresponding author.

## Data Availability

The data that support the findings of this study are available from the corresponding author upon reasonable request.
